# Editorial Commentary on the Special Issue “Antioxidant Therapy for Cardiovascular Diseases”—Cutting-Edge Insights into Oxidative Stress and Antioxidant Therapy in Cardiovascular Health

**DOI:** 10.3390/antiox13091034

**Published:** 2024-08-26

**Authors:** Guim Kwon, K. Michael Gibson, Lanrong Bi

**Affiliations:** 1 Department of Pharmaceutical Sciences, Southern Illinois University Edwardsville, Edwardsville, IL 62026, USA; 2 Department of Pharmacotherapy, Washington State University, Pullman, WA 99164, USA; mike.gibson@wsu.edu; 3 Department of Chemistry, Michigan Technological University, Houghton, MI 49931, USA

Recent advances in cardiovascular research have increasingly emphasized oxidative stress as a central mechanism in the pathogenesis and progression of cardiovascular diseases. Thus, this Special Issue on “Antioxidant Therapy for Cardiovascular Diseases” brings together cutting-edge studies that explore the complex interactions between oxidative stress, inflammation, and cardiovascular pathology alongside innovative antioxidant strategies that hold promise for mitigating these detrimental effects and improving patient outcomes.

Recent research continues to unravel the intricate connections between oxidative stress and various cardiovascular [1,2] and neurological complications [3], crucial in determining disease progression and patient outcomes. Notably, Bi’s and Shan’s team contribute significantly to this understanding by investigating brain-derived extracellular vesicles (EVs) from hypertensive rats, revealing that these EVs are potent inducers of neuroinflammation and oxidative stress [4], elevating pro-inflammatory cytokines and mitochondrial reactive oxygen species (mtROS) in the brain. This novel discovery underscores the potential of targeting EV-mediated oxidative stress at the molecular level to alleviate cardiovascular complications in hypertensive patients.

Complementing this, Shi and Meng’s team explores the cardioprotective properties of dihydromyricetin (DHY), a flavonoid known for its potent antioxidative effects, particularly in diabetic cardiomyopathy [5]. Their research demonstrates that DHY significantly enhances cardiac function and reduces myocardial injury by activating Sirtuin 3 (SIRT3), an essential mitochondrial protein involved in cellular stress responses. By modulating SIRT3, DHY effectively attenuates oxidative stress and inflammation, offering a promising therapeutic avenue for managing cardiovascular damage linked to diabetes, aligning with the growing interest in targeting mitochondrial pathways for therapeutic interventions [6,7].

Adding to these insights, a study led by Bourgonje et al. within the Prevention of Renal and Vascular End-stage Disease (PREVENT) cohort emphasizes the value of redox-related biomarkers in predicting cardiovascular (CV) events and all-cause mortality [8]. This large-scale study assessed homocysteine, gamma-GT, HDL cholesterol, bilirubin, and protein-adjusted free thiol (R-SH) levels in 5955 subjects, finding that protein-adjusted R-SH and homocysteine levels were significantly associated with the risk of CV events in men. By contrast, R-SH and HDL cholesterol levels were linked to all-cause mortality. In women, bilirubin and homocysteine levels were similarly predictive. These findings highlight the significance of R-SH levels in cardiovascular risk assessment and their potential for therapeutic intervention, reaffirming the role of oxidative stress-related biomarkers in clinical practice [9].

The studies featured in this issue underscore the importance of targeting specific molecular pathways to combat cardiovascular diseases effectively. Nguyen et al. provide a detailed analysis of how phosphate transporters, such as Type III Na+-dependent phosphate cotransporters and mitochondrial phosphate carriers, contribute to vascular calcification, a process closely associated with oxidative stress [10]. Their research suggests that inhibiting these transporters could prevent or mitigate vascular calcification, addressing a critical factor in the progression of atherosclerosis and other cardiovascular conditions. This aligns with recent advancements in understanding the role of vascular smooth muscle cells in calcification and the potential of targeting cellular phosphate handling as a therapeutic strategy [11,12].

Similarly, Han et al. explore the effects of lotus bee pollen extract (LBPE) on cardiomyocyte hypertrophy [13]. They reveal that LBPE can suppress the JAK2/STAT3 signaling pathway, which is crucial for regulating inflammatory responses and cell growth. By inhibiting this pathway, LBPE reduces oxidative stress and inflammation, preventing cardiomyocyte hypertrophy. These findings position LBPE as a promising natural therapeutic agent and contribute to a broader understanding of how targeting specific inflammatory pathways can provide cardioprotection.

The recurring theme of inflammation as a driving force in cardiovascular disease is strongly reflected in the studies presented in this issue [14]. Chen et al.’s investigation into brain-derived EVs from hypertensive rats reveals that these vesicles induce oxidative stress and trigger significant neuroinflammation, which can exacerbate cardiovascular conditions [4]. This study adds to the growing body of evidence suggesting that inflammation and oxidative stress are interlinked processes contributing to cardiovascular pathology, highlighting the potential of targeting these inflammatory pathways to improve cardiovascular health, particularly in hypertensive patients. The anti-inflammatory properties of LBPE, as demonstrated in Han et al.’s research [13], further highlight the benefits of integrating anti-inflammatory strategies with antioxidant therapies for more comprehensive protection against the multifactorial nature of cardiovascular diseases [14,15].

Recent research has brought significant attention to the role of sirtuins, particularly SIRT1 [16] and SIRT3 [17], in cardiovascular health. Chen et al. study on DHY’s activation of SIRT3 provide robust evidence that modulating these proteins can confer cardioprotection by reducing oxidative stress and inflammation [5]. This finding is consistent with studies suggesting sirtuins play a crucial role in mitochondrial biogenesis, metabolism, and cellular survival, making them attractive targets for therapeutic intervention in cardiovascular diseases. Leal et al.’s exploration of SIRT1 further underscores its importance in lipid metabolism and cardiovascular health [18], showing that lifestyle modifications such as energy restriction, alongside pharmacological interventions like atorvastatin, can enhance SIRT1 levels, improving HDL function and reducing LDL cholesterol levels. These findings support the therapeutic potential of sirtuin modulation [19] and highlight the importance of personalized interventions considering an individual’s metabolic profile and genetic predispositions.

In parallel, Leszto et al.’s investigation into selenium’s role in redox modulation adds a new dimension to our understanding of antioxidant therapy [20]. Their review highlights selenium’s ability to protect myocardial cells from oxidative damage, a critical factor in preventing the progression of cardiovascular diseases. However, it also calls for a nuanced approach to selenium supplementation [21,22], considering patient-specific factors to maximize benefits and minimize risks.

The collective findings presented in this Special Issue strongly advocate for integrating personalized medicine into cardiovascular health management. As research continues to elucidate the complex interactions between oxidative stress, inflammation, and cardiovascular disease, it becomes increasingly clear that therapeutic strategies must be tailored to the individual [23]. By leveraging advancements in biomarker research, genetic profiling, and molecular pathway analysis, healthcare providers can develop more targeted and effective antioxidant therapies.

The future of cardiovascular medicine will undoubtedly be shaped by these personalized approaches, ensuring that each patient receives care that is both specific to their needs and grounded in the latest scientific evidence. This Special Issue offers a comprehensive overview of the latest progress in understanding the role of oxidative stress and inflammation in cardiovascular diseases, advancing knowledge of the underlying mechanisms, and identifying promising therapeutic targets that could revolutionize treatment. As exploration of these pathways continues, the potential for translating these findings into clinical practice becomes increasingly evident, marking a significant milestone in the ongoing quest to develop effective, personalized antioxidant therapies and setting the stage for future innovations in cardiovascular disease management.
